# Uterine Natural Killer Cells: Functional Distinctions and Influence on Pregnancy in Humans and Mice

**DOI:** 10.3389/fimmu.2017.00467

**Published:** 2017-04-24

**Authors:** Louise M. Gaynor, Francesco Colucci

**Affiliations:** ^1^Centre for Trophoblast Research, University of Cambridge, Cambridge, UK; ^2^Department of Obstetrics and Gynaecology, National Institute for Health Research Cambridge Biomedical Research Centre, University of Cambridge School of Clinical Medicine, Cambridge, UK

**Keywords:** uterine natural killer cells, uterine innate lymphoid cells, placenta, pregnancy, arterial remodeling, trophoblast

## Abstract

Our understanding of development and function of natural killer (NK) cells has progressed significantly in recent years. However, exactly how uterine NK (uNK) cells develop and function is still unclear. To help investigators that are beginning to study tissue NK cells, we summarize in this review our current knowledge of the development and function of uNK cells, and what is yet to be elucidated. We compare and contrast the biology of human and mouse uNK cells in the broader context of the biology of innate lymphoid cells and with reference to peripheral NK cells. We also review how uNK cells may regulate trophoblast invasion and uterine spiral arterial remodeling in human and murine pregnancy.

## Introduction

CD56^superbright^ uterine natural killer (uNK) cells are present in human endometrium prior to the initiation of pregnancy, and markedly expand and become progressively more granulated during the progesterone-dominated secretory phase after ovulation and throughout the first trimester (1–3). uNK cells within the decidua have a distinct phenotype compared to peripheral blood NK (pbNK) cells and share features of both CD56^bright^ and CD56^dim^ pbNK subsets (Table 1). Similarly to CD56^bright^ pbNK cells, uNK preferentially produce cytokines and are poorly cytotoxic, despite their abundant intracellular granules containing granzymes, granulysin, and perforin (4–9). Killer-cell immunoglobulin-like receptors (KIR) and natural killer group 2 (NKG2)A/C/E receptors, which recognize trophoblast MHC class I human leukocyte antigen (HLA)-C and HLA-E, respectively, are expressed at higher levels among uNK than their pbNK cell counterparts, and are skewed toward recognition of their respective ligands (8, 10, 11). All human decidual uNK cells are CD49a^+^, also known as very late antigen-1 (VLA-1) or integrin α_1_β_1_, and express CD69 (2, 12). uNK cells peak in frequency during the first trimester, before becoming progressively less granular and beginning to diminish in numbers midway through gestation, so that only small numbers are present at term (13, 14).

**Table 1:** **Characterization of human natural killer (NK) cells in peripheral blood and decidua**.

Characteristic	Peripheral blood NK (pbNK) cells		Uterine NK (uNK) cells
	CD56^bright^ CD16^**−**^	CD56^dim^ CD16^**+**^	CD56^superbright^ CD16^**−**^
% of total	~ 5–30% circulating lymphocytes (15)		≥70% leukocytes in first trimester (2)
% of total NK cells	10% (16)	90% (16)	80% (2)
CD94	CD94^bright^ (17)	50% CD94^dim^ (18)	CD94^bright^ (18)
Natural killer group 2 (NKG2)A/C/E	+ (8)	+ (8)	++ (8, 11)
NKG2D	+ (19)	+ (19)	++ (20)
Killer-cell immunoglobulin-like receptors (KIR)	− (21)	+ (21)	++ (8, 22)
NKp46	+ (23)	+ (23)	+ (4)
CD9	− (8)	− (8)	+ (8)
CD49a	− (12)	− (12)	+ (12)
CD57	− (24)	60%+ (24)	− (2)
CD69	− (25)	− (25)	40%+ (2)
Cytokine production	+++ (26)	+ (26)	+++ (4, 5)
Cytotoxicity	− (21, 24)	+++ (21, 24)	− (6)

**Figure 1:**
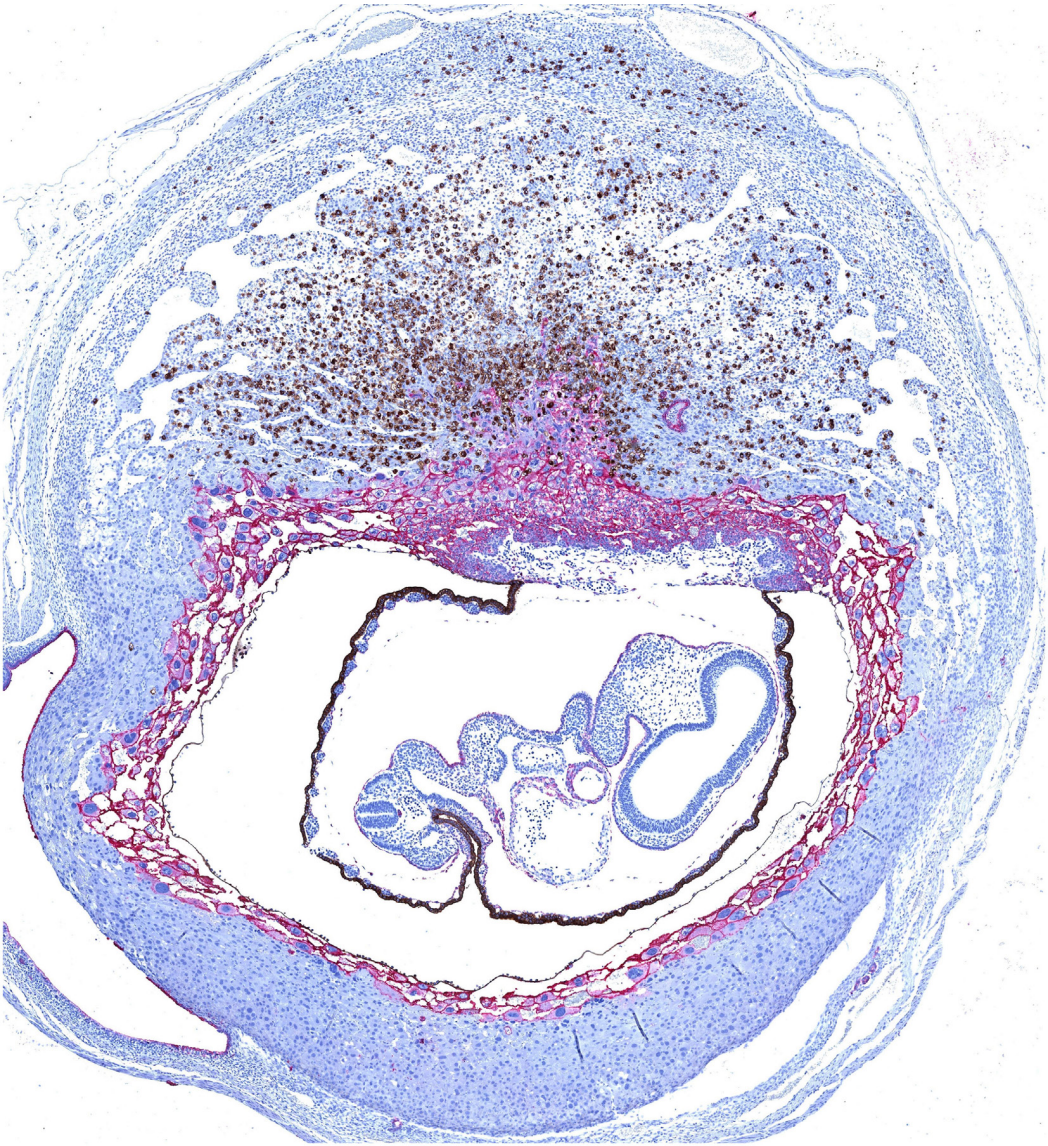
**Dual immunohistochemical staining of *Dolichos biflorus* agglutinin (DBA)^+^ uterine natural killer (uNK) cells and trophoblast in a mouse implantation site at mid-gestation**. Trophoblast (shown in *pink*) migrate centrally into the decidua to form an ectoplacental cone, which is surrounded by a layer of moderately invasive trophoblast giant cells expressing MHC class I. Mature uNK are abundant throughout the decidua basalis, and in a lymphocyte-rich accretion at the uppermost pole of the implantation site, known as the mesometrial lymphoid aggregate of pregnancy. Interactions between uNK Ly49 receptors and trophoblast MHC class I can modulate the activity of DBA^+^ uNK (shown in brown) and DBA^−^ uNK cells, and impact on their production of angiogenic factors and interferon (IFN)-γ respectively. Reproduced from Moffett and Colucci (39) with permission.

In comparison to humans, two functionally disparate populations of uNK cells have been identified in mice, which are distinguished in most studies to date by their reactivity to *Dolichos biflorus agglutinin* (DBA). Gene expression studies show that DBA^+^ uNK cells predominantly express transcripts for angiogenic factors, whereas interferon (IFN)-γ transcripts dominate in the DBA^−^ subset (27). Murine uNK do not begin to mature into large, granulated lymphocytes until blastocyst implantation, and they acquire reactivity to DBA after g.d. 5 alongside their increase in granularity (28, 29). As in humans, murine uNK cells are poorly cytotoxic despite containing granules encasing perforin and granzymes (30–32). At the mesometrial pole of the implantation site and adjacent to the decidua basalis, a lymphocyte-rich accretion of leukocytes composed largely of uNK cells, macrophages, and dendritic cells develops (29, 33, 34). This mesometrial lymphoid aggregate of pregnancy (MLAp) is a feature of pregnancy unique to rodents, which is established by g.d. 8. Mature uNK cells are most abundant throughout the decidua basalis and MLAp approximately halfway through gestation (Figure 1) (28, 29, 35). uNK undergoing apoptosis begin to appear from mid-gestation onwards and are highly prevalent by g.d. 12 (28, 35). Expression of lectin-like Ly49 receptors, which recognize MHC class I, is higher among uNK than peripheral (pNK) cells and, as in humans, some receptors are mildly skewed toward recognition of trophoblast MHC ligands (36, 37). uNK in mice also express killer-cell lectin-like receptor G1 (KLRG1) more highly than their pNK cell counterparts, indicating a more mature phenotype (36, 38). The features of murine uNK cells are summarized in Table 2.

**Table 2:** **Characterization of murine natural killer (NK) cells in spleen and decidua**.

Characteristic	Peripheral NK (pNK) cells	Uterine natural killer (uNK) cells
% of total lymphocytes	~ 2%	~ 30% at mid-gestation (40)
CD94	+ (41)	No published evidence
NKG2A/C/E	+ (41, 42)	No published evidence
NKG2D	+ (43)	+ (44)
Ly49s	+ (45)	++ (36)
NKp46	+ (46)	+ *Dolichos biflorus* agglutinin (DBA)^−^, ++ DBA^+^ (47)
CD49a	− (48)	~ 75% CD49a^+^ CD49b^+/−^ (33)
CD49b	+ (49)	~ 25% CD49a^−^ CD49b^+^ (33)
CD69	− (50)	++ (38, 51)
Killer-cell lectin-like receptor G1	+ (52)	++ (36)
Cytokine production	+ (53)	+ (54, 55)
Cytotoxicity	+ (56)	− (57)

The relatively recent designation of CD49a as a marker of tissue residency and its inclusion in the cytometric analysis of uterine lymphocytes alongside common NK cell markers such as CD49b (DX5) has enabled the redefinition of murine uNK subsets (33, 48). uNK cells in mice can now be classified as CD49a^+^ DX5^+/−^ uterine tissue-resident NK (trNK) cells and CD49a^−^ DX5^+^ uterine conventional NK (cNK) cell populations (33, 48, 58, 59), which will be described in greater depth later in this review. DBA reactivity is strongest on uterine CD49a^+^ trNK, and is weak on DX5^+^ uterine cNK (40, 58). As in DBA^+^ uNK, decidual CD49a^+^ DX5^+/−^ trNK cells produce less total IFN-γ at mid-gestation than CD49a^−^ DX5^+^ cNK cells, which further supports the correlation between CD49a and DBA reactivity (27, 58, 59). Although the correlation between CD49a and DBA co-expression is not sufficiently clear-cut to consider DBA as a specific marker of uterine trNK cells, it does enable some reconsideration of historical histological studies.

**Figure 2:**
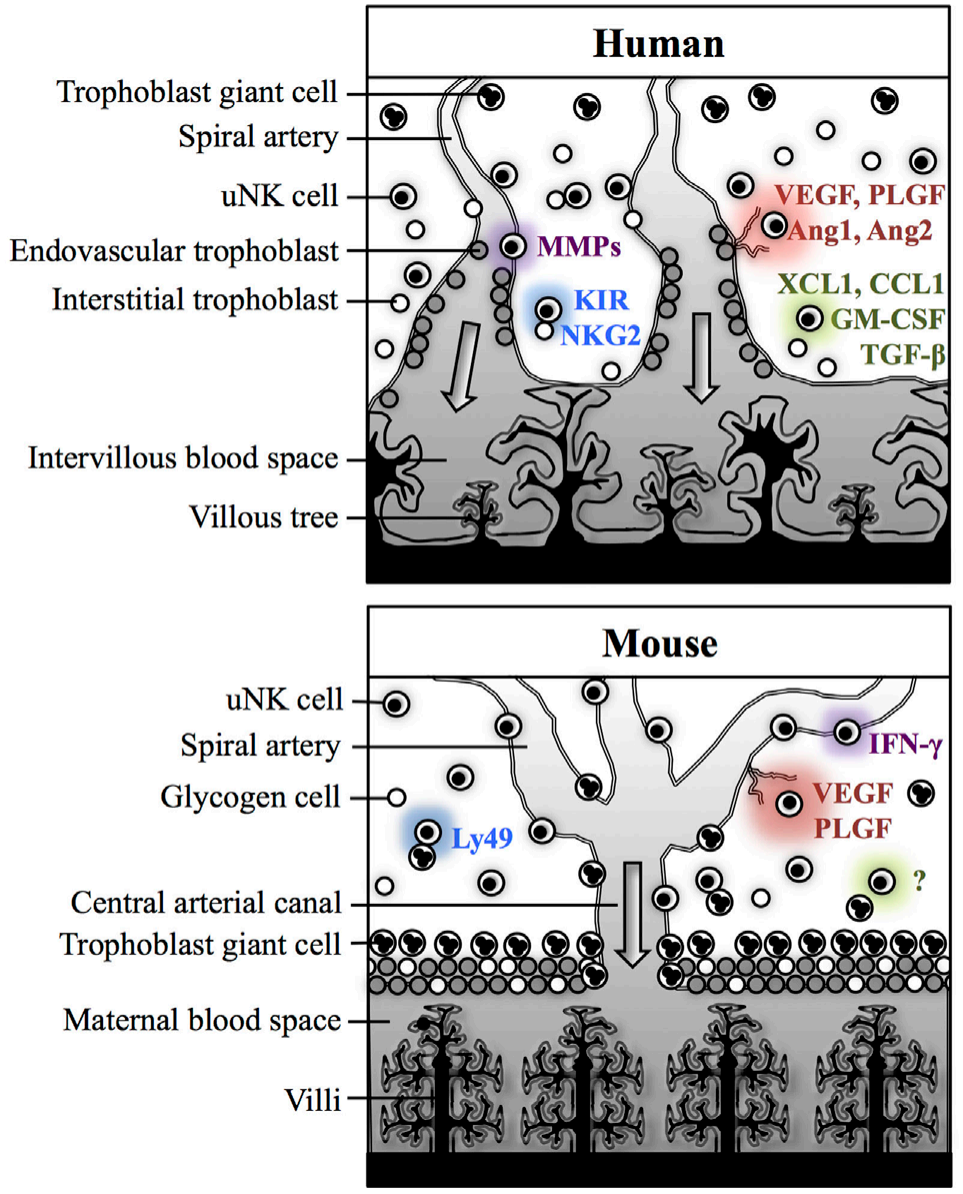
**Physiological processes of pregnancy within the decidua in human and mice**. In both humans and mice, uterine natural killer (uNK) cells are thought to contribute to spiral arterial remodeling (*purple*), angiogenesis (*red*), and control of trophoblast invasion (*green*). Interactions between uNK receptors and trophoblast MHC class I antigens may modulate uNK cell activity (*blue*). In humans, uNK cells may contribute directly to decidua-associated vascular remodeling through secretion of matrix metalloproteinases (MMPs). Human uNK may also influence trophoblast-mediated vascular remodeling through secreting factors which enhance extravillous trophoblast (EVT) invasion [XCL1, CCL1, granulocyte-macrophage colony-stimulating factor (GM-CSF)] or suppress EVT migration [transforming growth factor (TGF)-β]. uNK cells in humans also secrete several angiogenic factors including vascular endothelial growth factor (VEGF), placental growth factor (PLGF), Ang1, and Ang2. Their activity may be modulated by killer-cell immunoglobulin-like receptors (KIR) and natural killer group 2 (NKG2) receptors, which recognize human leukocyte antigen class I ligands expressed by EVT. In mice, IFN-γ secreted by *Dolichos biflorus* agglutinin (DBA)^−^ uNK cells is essential for decidual vascular remodeling. DBA^+^ uNK cells are predominantly responsible for producing angiogenic factors, including VEGF and PLGF. Evidence from studies in rats and mice suggests that uNK cells primarily suppress trophoblast motility, but the mechanisms for this are not currently understood. Murine uNK cell function can be modulated by Ly49 receptors, which recognize MHC class I expressed by trophoblast giant cells.

Despite numerous anatomical and physiological differences between murine and human pregnancies, the functions and regulation of uNK cells are reasonably comparable between these species. In both species, uNK contribute to fundamental physiological processes of pregnancy within the decidua, but there are key differences in how these effects are mediated (Figure 2). Human uNK assist in the initial stages of decidua-associated vascular remodeling and control the depth of invasion of extravillous trophoblast (EVT), which are responsible for the majority of arterial transformation in human pregnancy. Comparatively, murine uNK are composed of two subsets, with largely differing roles. uNK-derived IFN-γ is essential for remodeling of the decidual vasculature in mice, whereas the contribution of trophoblast is relatively insignificant and, indeed, rodent uNK predominantly suppress trophoblast invasion. In both species, uNK produce angiogenic factors, but in mice this is predominantly mediated by the DBA^+^ subset. As such, considering the broader themes of the decidual adaptations to pregnancy, mice provide a useful animal model in which to study reproductive immunology.

## Innate Lymphoid Cells

Natural killer cells are the most abundant and well-characterized subset of innate lymphoid cells (ILCs), which comprise lymphocytes belonging to the innate arm of the immune system exhibiting features of both innate and adaptive immunity (60, 61). ILCs are an important component in the immune response to a wide range of pathogens, particularly at epithelial barrier surfaces. They also contribute to tissue and metabolic homeostasis and have been implicated in the pathogenesis of cancer and inflammatory diseases. Features common to all ILCs are the absence of recombination activating gene (RAG)-dependent antigen-specific receptors, absence of myeloid lineage markers, and a lymphoid cellular morphology. Based upon this classification, ILCs can be broadly categorized into three major groups according to their development, cell surface markers, and functions (62, 63).

Our understanding of the origins and functions of ILCs is rapidly evolving, and the identities of disparate ILC populations are becoming increasingly apparent (64–67). While the pathways of ILC development in mice have been well defined, ILC differentiation in humans has yet to be determined. There is also a degree of phenotypic plasticity among ILCs, indicating that ILCs are capable of adapting their identities and functions *in vivo* in response to other immune cells and secreted factors in the local environment.

Functional comparisons have been made between ILCs and T cells, as the stimuli and cytokine profiles of ILC1s, ILC2s, and ILC3s are analogous to those of the T_H_ cell subsets T_H_1, T_H_2, and T_H_17, respectively. Group 1 ILCs comprise *bona fide* helper-like ILC1s and NK cells. NK cells can be further subdivided into cNK and trNK subsets that differ in their phenotype, function, and development. cNK cells are the only ILC population to exhibit cytotoxicity mediated by exocytosis of cytotoxic granules containing perforin and granzymes, similarly to CD8^+^ cytotoxic T-lymphocytes (60).

## Uterine ILCs

Since the amalgamation of diverse innate lymphocyte populations into the ILC family, there has been considerable focus on determining the distribution and biological significance of these cells *in vivo* (68). Other than uNK cells, first defined in 1991, ILC1s and ILC3s have also been identified in human decidua (2, 33, 69, 70). Uterine ILC3s (uILC3s) were initially classified as stage 3 uNK cell progenitors based upon their CD34^−^ CD117^+^ CD94^−^ CD56^+^ KIR^−^ phenotype. These cells produced interleukin (IL)-22 and expressed *RORC* and *LTA*, encoding the transcription factor RORγ and lymphotoxin (LT)-α, respectively, which makes them indistinguishable from uILC3s (62, 69). The presence of human uILC3s and lymphoid tissue inducer (LTi)-like uILC3s has since been confirmed in accordance with currently accepted ILC definitions (33, 70). However, it has since been proposed that a population of CD34^+^ CD122^+^ CD309^−^ lymphoid-like cells in human decidua represent NK-committed decidual hematopoietic progenitor cells (HPCs) (71). If these cells can be more definitively characterized as such, through detection of multiple co-expressed cell surface markers and transcription factors, it is likely that the population described by Male et al. were a heterogeneous mix of stage 3 uNK cell precursors and uILC3s. A proportion of uILC3s have been shown to differentiate to stage 3-like CD117^+^ CD56^+^ CD94^+^ uNK cells upon *in vitro* culture with IL-15 (70). A similar report indicates that uILCs can differentiate into stage 4 CD117^−^ CD56^+^ CD94^+^ uNK cells *in vitro*, further suggesting that uNK cell precursors were present (69). In view of the recent finding that tonsillar ILC3s can differentiate to stage 4 CD94^+^ CD56^bright^ NK cells upon aryl hydrocarbon receptor (AhR) silencing *in vitro*, it would also be interesting to ascertain whether AhR is expressed by uILC3s and whether its manipulation is similarly able to induce differentiation to an NK cell phenotype (72).

All groups of ILCs are present in the uteri of virgin and pregnant mice (33). Uterine trNK cells were initially considered to develop independently of the transcription factors nuclear factor, interleukin-3 regulated (Nfil3) and T-box transcription factor Tbx21 (T-bet), but their dependency on Eomesodermin (Eomes) was not ascertained. As such, it was not possible to deduce their identity as belonging to an NK cell or *bona fide* ILC1 lineage (48). Similarly to those in the salivary gland, trNK cells in the uterus do express Eomes and, together with uterine cNK cells and Eomes^−^ uILC1s, they are found throughout the decidua and myometrium during pregnancy. uILC1s can produce tumor necrosis factor (TNF)-α and IFN-γ but, owing to the fact that uILC1-sufficient *Nfil3*^−/−^ females exhibit poor decidual vascular remodeling, the contribution of uILC1-derived IFN-γ to vascular modification is seemingly negligible (33, 58, 59). uILC2s, uILC3s, and LTi-like uILC3s are found only in the myometrium and in the MLAp (33). The MLAp is of unknown function but, since it is traversed by branches of the uterine artery, it is possible that it exerts some effect on the perfusion of individual implantation sites through leukocyte-mediated modification of vessels proximal to the spiral arteries (73, 74). Unlike lymph nodes, MLAp formation does not depend on LTα and LTβ-receptor signaling, making a role for LTi-like uILC3s in MLAp development unlikely (35, 75). Whether uILC2s and uILC3s and their derived cytokines, IL-5, IL-13, IL-17, and IL-22 participate in local immune regulation or tissue remodeling is currently unknown (33).

## NK Cell Development

Murine cNK cells arise from NK progenitors (NKP), which represent the first of six defined stages of murine NK cell development (76–78). In contrast to helper-like ILCs, cNK cells develop independently of IL-7 and become CD127^−^ CD122^+^ (IL-2Rβ; IL-15Rβ) at the NKP stage. IL-15 signaling is essential for the differentiation of NKPs to immature NK (iNK) cells (79–81). Subsequently, iNK cells acquire functionally modulatory receptors such as NK1.1, NKp46, Ly49, and NKG2 receptors. Expression of the integrin CD49b (DX5) denotes the transition of iNK cells to a mature phenotype, which correlates with the development of functional competence such as IFN-γ production and cytolytic potential (82, 83). Three further stages of maturation can be defined by the differential expression of CD27 and CD11b, which culminate in the development of terminally mature NK cells expressing KLRG1 and CD43 (52, 84).

However, NK cell development can occur *via* alternative pathways. NK cells of thymic origin have been identified, which depend upon the GATA-binding protein-3 (GATA-3) transcription factor and IL-7 signaling. These cells appear phenotypically immature compared to cNK cells, but are more effective cytokine producers (85). More recently, Nfil3-independent NK cells have been described in skin, uterus, and salivary glands, which all express the integrin CD49a as a marker of tissue residency but which differ in their dependency on T-bet (33, 48, 86–88). However, since the population originally classified as CD49a^+^ DX5^−^ hepatic trNK cells does not express the transcription factor Eomes, it is more appropriate to consider these as hepatic ILC1s. These exhibit a broader cytokine profile than cNK and highly express TNF-related apoptosis-inducing ligands (TRAIL), which confer potential to induce apoptosis (48, 89, 90).

Ontogenesis of human NK cells is broadly analogous to that in mice, but there are notable differences in the sequence and anatomical sites of each developmental stage. Human NK cells arise from bone marrow (BM)-derived CD34^+^ HPCs. Although elusive until recently, NK lineage-restricted progenitors have been identified in adult and fetal bone marrow, fetal liver, and adult tonsils (91). Evidence suggests that CD34^dim^ pro-NK cells are exported from BM comparatively early and home to secondary lymphoid tissues where they continue to differentiate (92). Five continuous stages of human NK cell development have been characterized in lymph node and tonsil. IL-15 acts on stage two pre-NK cells to support their transition to stage three. Human NK cells do not begin to express receptors for class I HLA antigens, including KIR and CD94/NKG2 dimers, until they reach a mature CD56^bright^ phenotype (17). At this stage, human NK cells are competent cytokine producers, which either remain *in situ* or terminally differentiate in peripheral blood to acquire cytotoxic potential as CD56^dim^ CD16^+^ NK cells (21, 26, 93).

The transcriptional control of human NK cell development has not been delineated clearly, but *GATA-3* transcripts are abundant in stage 3 NK cells, and T-bet and Eomes are highly expressed in stage four and stage five NK cells (17, 94). However, as in mouse, subpopulations of CD49a^+^ trNK cells have been identified in uterine endometrium and liver (59, 95). A subpopulation of CD127^−^ CD56^+^ Eomes^+^ tonsillar and intestinal intraepithelial ILC1s are phenotypically and functionally resemblant of NK cells, but their murine counterparts develop independently of IL-15 (96). As such, it is possible that these, and perhaps other ILC1s, arise from pre-NK cells, and are more closely developmentally linked to NK cells than we currently appreciate.

## Origin of uNK Cells

Uterine natural killer cells account for over 70% of decidual leukocytes in the first trimester of human pregnancy and for approximately 30% of lymphocytes in murine decidua at mid-gestation (2, 40). The origin of these distinct and specialized NK cells has been a subject of investigation for over 30 years, but it is becoming increasingly accepted that uNK cells are likely to be a heterogeneous population arising from *in situ* progenitors and from homing of NKPs and/or pNK cells (97).

When mice were lethally irradiated in the presence of a protective lead shield covering one uterine horn, and subsequently rat BM cells were adoptively transferred, only uNK cells of rat origin could be identified in the irradiated uterine tissue, indicating that peripherally derived NKPs contribute to the generation of uNK cells (98). This is supported by observations that uteri from NK-sufficient mice are devoid of uNK cells when engrafted into NK-deficient hosts (99). Leukocytes of donor origin can be found in both murine and human decidua, following experimental transgenic labeling of BM cells and hematopoietic stem cell transplantation (HSCT) respectively, which suggests that decidual leukocytes are derived, at least in part, from BM HPCs *in vivo* (100, 101). A very small population of stage 3 NK precursors in peripheral blood, which are capable of maturing to stage 4 cells in the presence of IL-15, also raises the possibility that NK precursors home to the uterus and differentiate to mature uNK cells *in situ* (69). As pbNK cells can be induced to acquire phenotypic and functional attributes of uNK cells under the influence of hypoxia, transforming growth factor (TGF)-β, and demethylating agents, it is also possible that some uNK cells develop as a result of pbNK cell recruitment (102).

However, in the study by Peel and Stewart, no uNK cells could be detected in the irradiated uterine horn in half of the mice which had retained functional BM as a result of lead shielding of their legs during irradiation. This suggests that uNK cell precursors present in uterine tissue prior to irradiation were either destroyed or rendered incapable of proliferation, and that recruitment of circulating NKPs was insufficient to restore the uNK cell population (98). The proposed NK-committed decidual HPCs identified by Vacca et al. can differentiate to mature uNK cells in the presence of IL-15, which is expressed abundantly in first trimester decidua and placenta (71, 103). The presence of *in situ* HPCs would also account for the CD56^+^ NK cells detected in human endometrial tissue which had been xenografted into hormone-treated immunodeficient mice (104). NK cell development from resident hematopoietic progenitors has also been documented in mice (105). Taken together, it seems probable that uNK cells arise from proliferation of peripherally derived HPCs and/or NK precursors which have homed to the pregravid uterus, but a potential contribution by pbNK cells which undergo phenotypic adaptation *in situ* cannot be discounted.

## Effector Functions of NK Cells

Soluble factors secreted by other leukocytes can stimulate cytokine production by NK cells, which provides a means by which these immune cells can indirectly interact with each other and reciprocally induce effector functions. NK cells are responsive to a number of cytokines released by monocytes, including IL-1, IL-10, IL-12, IL-15, and IL-18, and T_H_ lymphocytes, including IL-2 and IL-21. These induce production of key NK cell-mediated cytokines such as IFN-γ, granulocyte-macrophage colony stimulating factor (GM-CSF), TNF-α, and macrophage inflammatory protein (MIP)-1 (106). Of these, IFN-γ has the most diverse immunomodulatory roles and promotes T_H_1 cell differentiation, activation of macrophages and enhancement of antigen presentation *via* upregulation of class I and class II MHC molecules; all of which cumulatively contribute to antimicrobial, antiviral, and anti-tumor immunity (107).

All mature murine cNK cells have the capacity to produce cytokines and mediate perforin-dependent cytotoxicity. Distinct tissue-specific NK cell subpopulations display variation in functionality, such that salivary gland trNK cells only induce TRAIL-dependent cytolysis and uNK cells are weakly cytotoxic under physiological conditions (57, 87). In humans, CD56^dim^ CD16^+^ NK cells contain lytic granules, and are less effective cytokine producers and express KIR at far higher frequencies than their CD56^bright^ CD16^−^ counterparts (21, 26). That the CD56^dim^ CD16^+^ subset accounts for 90% of circulating NK cells emphasizes the importance of HLA class I recognition as a means of immunosurveillance by pNK cells. Indeed, the absence of NK cells *in vivo* enhances susceptibility to viral infections and metastatic progression of malignant tumors (108–110).

Natural killer cells express a broad repertoire of modulatory receptors, of which many are common to both human and mouse. Among these are the activating receptors NKp46, which recognizes viral hemagglutinins, NKG2D which binds cellular stress-induced ligands, and CD16, which mediates antibody-dependent cellular cytotoxicity (ADCC) in response to immunoglobulin G (IgG) (111, 112). The induction of cytotoxic effector responses is tightly regulated and, with the exception of CD16, requires the synergistic input of signaling *via* two activating receptors, reduced inhibition and/or the presence of stimulatory cytokines (113). As many inhibitory NK cell receptors recognize MHC class I ligands, reduced inhibition predominantly occurs in the context of downregulation of MHC class I molecules by virally infected and malignant cells.

The probable roles of uNK in both human and mouse are the production of cytokines, chemokines and angiogenic factors, which may mediate the key physiological processes required for successful pregnancy, discussed in greater depth later in this review (Figure 2). Comprehensive gene expression analyzes have demonstrated the extent to which human NK cells in the uterus functionally and phenotypically differ from those in peripheral blood (4, 8). Although uNK cells are phenotypically and functionally distinct from pNK cells, their activity can be similarly modulated through interactions with soluble factors and cell-bound ligands, including MHC class I.

## NK Cell Recognition of MHC Molecules

Recognition of class I MHC is mediated by KIR in humans, Ly49 receptors in mice, and by CD94/NKG2 heterodimers in both species (114). KIRs are highly polymorphic receptors encoded within the leukocyte receptor complex (LRC) on chromosome 19, which bind to HLA class I molecules (115). Sixteen *KIR* genes have been identified and, for each, between 18 and 112 alleles are currently known (116, 117). Fourteen of these genes encode functional receptors for classical HLA, of which six are inhibitory and eight are activating.

*KIR* genes can be grouped into two main haplotypes, termed *A* and *B*. With the exception of *KIR2DS4*, which is most commonly truncated, haplotype *A* encodes only inhibitory receptors whereas haplotype *B* contains genes for both inhibitory and activating KIR. The majority of KIR2D receptors exhibit binding specificity for one of two epitopes of all HLA-C allotypes, C1 and C2, which differ due to diallelic polymorphism at positions 77 and 80 of the α1 chain (116, 118). Binding affinities between KIR2DL and HLA-C molecules also influence functional responses, such that weak interactions induce less inhibition. *KIR A* haplotypes are typified by KIR2DL1, which binds C2 epitopes with high avidity, and KIR2DL3, which weakly binds C1 epitopes. Comparatively, *KIR B* haplotypes are characterized by an allotype of KIR2DL1 which binds C2 epitopes with low affinity, and KIR2DL2, which binds more strongly than KIR2DL3 to C1 epitopes (119).

The functionally analogous receptors for classical MHC class I molecules in the mouse are polymorphic lectin-like Ly49 receptors. These are encoded within the natural killer complex (NKC) on chromosome 6 and bind classical H-2 antigens. *Ly49* gene content varies considerably between strains, ranging from eight in BALB/c mice to 22 in non-obese diabetic (NOD) mice (120, 121). Ly49 receptors use the same signaling pathways as KIR, including intracytoplasmic immunoreceptor tyrosine-based inhibition motifs (ITIM) for inhibitory receptors and signaling through DAP12 for activating receptors (122–124). Nomenclature of *Ly49* genes, which are synonymous with killer cell lectin-like receptor subfamily A (*Klra*) genes, is non-descriptive and each receptor is designated a letter between A and X (125). The major Ly49 receptors in C57BL/6 (H-2^b^) and BALB/c (H-2^d^) strains and their respective ligands are summarized in Table 3. Of particular functional significance is the activating Ly49H receptor, which recognizes the murine cytomegalovirus (MCMV) m157 glycoprotein. BALB/c mice notably lack this receptor and, as such, are highly susceptible to MCMV, with high viral titers and increased mortality following infection (126, 127).

**Table 3:** **Ly49 receptors and their respective ligands in C57BL/6 and BALB/c mice**.

Ly49 receptor	Ligands	Notes
Ly49A	H-2D^d^, H-2D^b^, H2-M3 (128–130)	
Ly49C	H-2K^b^, H-2D^b^, H-2K^d^, H-2D^d^ (128, 129)	
Ly49D	H-2D^d^ (131)	Absent in BALB/c
Ly49G2	H-2D^d^ (128, 129)	
Ly49H	Murine cytomegalovirus m157 glycoprotein (127)	Absent in BALB/c
Ly49I	H-2K^b^, H-2K^d^ (128, 129)	Pseudogene in BALB/c

*Inhibitory receptors denoted in red, and activating receptors denoted in blue*.

In both humans and mice, recognition of non-classical MHC class I molecules is predominantly mediated by CD94/NKG2 heterodimers. Inhibitory CD94/NKG2A dimers signal *via* an ITIM-dependent pathway, whereas activating CD94/NKG2C and CD94/NKG2E associate with DAP12 (132–136). CD94/NKG2 dimers recognize HLA-E in humans and Qa-1^b^ in mice, which are expressed in complex with peptides derived from leader sequences of other MHC class I molecules (41, 42, 137, 138). As such, HLA-E and Qa-1^b^ provide an additional means by which aberrant MHC class I expression in diseased cells can be detected by NK cells. There are potentially some species-related differences in the expression profiles of these receptors, as human CD94/NKG2E exists only in an intracellular form, and cell surface expression of neither CD94/NKG2C nor CD94/NKG2E has been definitively detected in mice (41, 42, 134).

It has long been considered that acquisition of individual KIR and Ly49 receptors occurs stochastically, such that the co-expression frequencies of individual receptors do not deviate markedly from the product rule (139). This generates subsets of NK cells expressing anywhere between zero and the full complement of NK cell receptors for MHC class I herein referred to as NKRs. However, deviations in the NKR repertoire in accordance with the MHC environment indicate that there are some selective influences (140–143). Specific NKRs are downregulated in the presence of their cognate MHC ligands in a manner that is both MHC dose-dependent and reflective of receptor–ligand binding avidity (142, 144, 145). Refinement of the NKR repertoire is an important aspect of the adaptation of NK cells to their host environment, and is complementary to a process referred to as NK cell education, during which interactions with self-MHC calibrate NK cell responsiveness. Taken together, these processes may allow for selection of the most biologically useful and least self-reactive NK cell subsets *in vivo*.

## NK Cell Education

The concept of NK cell education, or “licensing,” was first proposed in 2005 when it was observed that cells expressing inhibitory NKRs for self-MHC are functionally more responsive than those that do not, both in terms of cytotoxicity and IFN-γ production. This was proposed as a mechanism for NK cell self-tolerance, so that uneducated cells lacking NKRs for self-MHC respond poorly to activating stimuli, such as cross-linking of activating receptors and MHC class I deficient cells. This negates the requirement for NKR-mediated counter-inhibition and reduces the potential for autoreactivity (140, 146). However, NK cell education is by no means an essential requirement for functionality, since responsiveness can be at least partially restored among uneducated NK cells in the presence of pro-inflammatory cytokines (140, 146–148).

The outcome of the educative process is that NK cells attain the capacity to respond to aberrant MHC class I expression. This occurs through “missing-self” recognition, which may result from the absence of self-MHC class I or in the presence of allogeneic MHC class I ligands. The latter effect can be harnessed for therapeutic benefit in HSCT, used in the treatment of hematological malignancies. NK cells from HLA haplotype-mismatched donors enhance graft tolerance in patients with acute myeloid leukemia and induce disease remission with protection against relapse (149). The only physiological situation in which allogeneic class I MHC is presented to a host is during pregnancy. uNK cells are a sufficiently distinct subset that their behavior cannot be effectively modeled on pNK cells, owing to significant phenotypic and functional differences. However, a wealth of evidence from human genetic association studies and mouse models suggests that uNK cell activity can be modulated through interactions with class I MHC from both parents, and that this has the potential to significantly impact on reproductive outcome (150).

## Regulation of Spiral Arterial Remodeling by uNK Cells

The placenta was originally thought to provide the means of “anatomical separation of fetus from mother,” enabling development of the semi-allogeneic fetus without maternal immune rejection (151). A modern view of immunogenetics of pregnancy proposes that in fact maternal NK cells regulate placentation and vascular remodeling through direct interactions with fetal trophoblast cells (150). Despite numerous anatomical and physiological differences between human and murine pregnancy, mice can provide a useful model in which to study trophoblast differentiation and immune regulation of placental development because both species exhibit hemochorial placentation, where placental trophoblast cells invade the maternal decidua and come into direct contact with maternal blood (152–154). Key features of human and murine pregnancy are summarized in Table 4.

**Table 4:** **Features of pregnancy in humans and mice (154–157)**.

Feature	Human	Mouse
Decidualization	Cyclical (approx. 28 days)	Post-implantation
	Prior to implantation	
Implantation	~ 6 days post-conception (p.c.)	Gestation day (g.d.) 4.5
Type of placentation	Discoid	Discoid
	Hemochorial	Hemochorial
	Villous	Labyrinthine
Placental development	First functional at 10 weeks p.c.	First functional at g.d. 10.5
	Growth continues until term	Maximal size at g.d. 16.5
Fetal growth	Disproportionate to placental growth in third trimester	Disproportionate to placental growth from g.d. 14.5
Fetal:placental weight ratio	Approx. 7.5:1	Approx. 15:1
Duration of gestation	~ 40 weeks	19–21 days

Remodeling of the maternal spiral arteries is an essential local vascular adaptation to pregnancy, which transforms the arteries supplying the feto-placental unit to large bore, high conductance vessels with non-turbulent flow (158). The initial stages of vascular transformation in humans occur during the secretory phase of the menstrual cycle and become more pronounced in early pregnancy independently of trophoblast invasion (159). The maternal vessels during these stages are closely apposed by leukocytes, particularly macrophages and uNK cells, which may contribute to this decidua-associated remodeling through secretion of proteolytic matrix metalloproteinases (MMPs). CD56^+^ cells have been shown histologically to express MMP-7, MMP-9, MMP-19, and MMP-23 (160–162). However, since CD56 is also expressed by endovascular EVT, it cannot be asserted from dual immunohistochemical staining alone that intramural and endovascular CD56^+^ MMP^+^ cells are uNK cells (159). Subsequent stages of remodeling in humans are dependent upon the deep invasion of EVT by interstitial and endovascular routes. Interstitial EVT migrate through the decidua and are thought to intravasate into the walls of the maternal spiral arteries to contribute to disorganization of the vascular smooth muscle (159, 163). Perivascular trophoblast may intravasate further into the vascular lumen to account for some of the endovascular trophoblast which migrates retrogradely along the lumen of the arteries (164). It is considered that EVT from both interstitial and endovascular routes become incorporated into the vascular wall and replace vascular smooth muscle cells (VSMCs) with fibrinoid material, which maintains the vessel in a dilated state and renders it incapable of vasoconstriction (165).

The contribution of trophoblast to decidual vascular transformation in mice is less well defined. Moderately invasive trophoblast giant cells (TGCs) associate with decidual vessels in their more distal segments, and line the arterial canals which supply the feto-placental unit (166). It seems likely that vascular modification in mice is predominantly mediated by other decidual cells, including uNK cells which become integrated into the vascular media (167). Indeed, it is well established that IFN-γ of uNK cell origin is essential for spiral arterial remodeling in murine pregnancy (54). NK cell-deficient mice consistently have defective decidual vascular remodeling, characterized by narrow vascular lumens, thick vascular walls, and retention of vascular smooth muscle actin (58, 168–170). Through utilizing alymphoid mice which were engrafted with BM from IFN-γ^−/−^ mice or severe combined immunodeficient (SCID) mice, which lack T- and B-lymphocytes, it has been elegantly and conclusively demonstrated that IFN-γ of uNK cell origin is essential for murine spiral arterial remodeling (54). NK cell-deficient mice also exhibit IFN-γ-dependent morphological abnormalities such as decidual hypocellularity and failure of MLAp formation, which can be restored through adoptive transfer of BM from C57BL/6 or SCID mice (54, 169, 171). The mechanisms by which IFN-γ mediates vascular remodeling have not been elucidated. Although murine uNK cells have not been reported to produce MMPs, decidual macrophages do produce MMP-9, and MMP-2 and MMP-9 expression can be observed throughout the decidua basalis and in close proximity of decidual arteries (172, 173). Since uNK cells produce IFN-γ and MIP-1α, which are key cytokines involved in macrophage activation, it is possible that uNK cells mediate vascular remodeling through stimulation of MMP production by macrophages (36, 107). Indeed, MMP-2 and macrophage-derived MMP-9 are essential in the pathogenesis of murine abdominal aortic aneurysms, in which pathological destruction of the aortic vascular media leads to extensive dilatation and risk of rupture (174).

More similarly to humans, modification of the spiral arteries in rats involves initial medial disorganization by uNK cells and subsequent destruction of the smooth muscle layer by interstitial and endovascular trophoblast, which invade deep into the decidua and myometrium (175, 176). This demonstrates that, even among species that exhibit hemochorial placentation, there is significant variability in the dependence upon trophoblast and uNK cells for transformation of the spiral arteries supplying the feto-placental unit.

## Regulation of Trophoblast Invasion by uNK Cells

Uterine natural killer cells may also contribute to modification of spiral arteries indirectly, through their influence on EVT. A recent study shows that human uNK produce the chemokines XCL1 and CCL1. The receptor for XCL1, XCR1, is expressed by several cell types in the placenta, including fetal endothelial cells and EVT. XCR1 is also expressed by decidual cells, including a small population of CD14^+^ macrophages. The CCL1 receptor, CCR8, has been identified on all decidual macrophages and on a small proportion of uNK (177). It has been determined by intracellular cytometry that uNK secrete GM-CSF, and the chemokines IL-8 and interferon-inducible protein (IP)-10 have been detected in supernatants of uNK cells *in vitro*. All of these factors enhance motility of primary trophoblast in cell migration and invasion assays (4, 177–180). Intracellular cytometry has since shown that macrophages are probably the predominant source of IL-8 among decidual leukocytes, although activated uNK cells were not assessed in this study (181). IL-8 stimulates production of MMP-2 and MMP-9 in a first trimester EVT cell line, which is suggestive of a mechanism by which leukocyte-derived factors may promote EVT-induced vascular remodeling (180). However, uNK also secrete TGF-β, which impairs the invasive properties of primary trophoblast *in vitro* (5, 182). As such, human uNK seemingly mediate a balance between enhancing and inhibiting EVT invasion, and alterations in their function may lead to placental pathology and associated disorders of pregnancy. Supernatants from IL-15-activated uNK cell isolates from women with high uterine artery resistance, which denotes incomplete arterial remodeling, do not effectively induce motility of a first trimester EVT cell line and apoptosis of VSMC and endothelial cell lines *in vitro* (183). While this likely indicates that uNK cell-derived factors contribute to vascular remodeling and modulating trophoblast migration *in vivo*, uNK cells were harvested in this study at 9–14 weeks gestation, when transformation of the decidual sections of the spiral arteries is advanced and uNK cell function is declining (184). Assessment of uNK function at an earlier gestational time-point would be more informative for understanding the relative contribution of uNK cells to these physiological processes.

Whereas human uNK cells have been demonstrated to both enhance and inhibit EVT invasion, evidence from studies in rats and mice suggests that uNK cells primarily suppress trophoblast motility. The onset of trophoblast invasion in both rats and mice was observed to correlate with the demise of uNK cells, at around g.d. 14 and was accelerated in NK cell-deficient and IFN-γ^−/−^ mice (176). This is seemingly dependent upon a profound deficit in uNK cell number and/or function, as no effect on depth of trophoblast invasion could be determined in a model of more subtle, MHC-dependent uNK inhibition (36). It has also been suggested that, through contributing to decidual angiogenesis, uNK cells contribute to increased oxygen tensions at the maternal–fetal interface, which prevents trophoblast adopting an invasive phenotype (175). This would most likely be mediated by murine DBA^+^ uNK cells, which are known to produce angiogenic factors including vascular endothelial growth factor (VEGF) and placental growth factor (PLGF) (27, 55, 185).

Human uNK also secrete several angiogenic factors including VEGF, PLGF, angiopoietin (Ang)1, and Ang2 (4, 5, 179). Production of all factors mentioned can be modulated through KIR/HLA interactions and by the activating receptors NKG2D, NKp30, NKp44, and NKp46 (4, 178, 179). Human trophoblast express ligands for NKp44, but not for NKG2D (4, 20, 179). However, since decidual stromal cells express ligands for NKp30 and NKG2D, it is likely that uNK cell function is also modulated through interactions with maternal tissues (4, 186). There is some evidence to suggest that ligation of NKp30 also induces production of IFN-γ, TNF-α, MIP-1α, and MIP-1β, but since uNK cells were stimulated in the presence of IL-2, the physiological significance of these results is questionable (187). Moreover, recent work suggests that the decidual microenvironment influences the expression of NKp30 and NKp44 splicing variants that may be responsible for decreased cytotoxicity and altered cytokine secretion of uNK cells compared to pbNK cells (188).

## Sequelae of Defective Placentation

Defective vascular remodeling, characterized by the absence of intramural EVT and retention of VSMCs, particularly within the myometrial segments of the spiral arteries, is a common pathologic feature in cases of pre-eclampsia, early miscarriage, unexplained stillbirth, and fetal growth restriction (FGR) (189–191). As such, these conditions may reasonably be considered as a spectrum of disorders that can arise from a common primary pathology and, collectively, they are often referred to as the Great Obstetric Syndromes. Some cases of recurrent miscarriage (RM) may also be caused by insufficient trophoblast invasion (192).

To date, many of the studies investigating impaired decidual vascular transformation in mice have focused on the causative mechanisms and histological features. Defective remodeling of spiral arteries does not spontaneously induce systemic hypertension in mice, but is linked to poor fetal growth, indicating that pre-eclampsia only occurs as a response to placental stress from underperfusion in humans (36, 193).

Human EVT invasion may also occur excessively when a blastocyst implants in poorly or non-decidualized tissue. Placenta accreta occurs due to pathological trophoblastic invasion of the myometrium, which most commonly occurs as a result of implantation at the site of uterine scar tissue from previous intrauterine surgery (194). Similar pathological features are observed in ectopic pregnancies, in which the thin wall of the Fallopian tube is commonly entirely infiltrated by EVT in the absence of decidual tissue (195). Given that trophoblast migration is enhanced in mice and rats depleted of NK cells, excessive invasion of EVT in non-decidualized tissue in humans is highly suggestive of a fundamental role for human uNK cells in regulating trophoblast invasion (175, 176).

## Immunogenetics of Trophoblast and uNK Cell Interactions

Uterine natural killer cell activity can be directly modulated through interactions with decidual stromal cells, uterine leukocytes, and invasive trophoblast. Of these, the regulation of uNK cell function by trophoblast has been particularly well explored, owing to the association between certain KIR/HLA interactions and disorders of pregnancy. Trophoblast cells express a distinct repertoire of HLA ligands in comparison to somatic cells. Syncytiotrophoblast, which directly contacts maternal blood, and villous cytotrophoblast are HLA negative (196). Invasive EVT express a unique combination of polymorphic HLA-C and oligomorphic HLA-E and HLA-G, but not HLA-A, HLA-B, or MHC class II (197). Each of the EVT HLA class I ligands is able to interact with uNK cell receptors (uNKRs), as outlined in Table 5. Soluble HLA-G is reportedly produced by trophoblast, and is suggested to modulate uNK cell activity (198, 199). However, assessment of the crystal structure of KIR2DL4 and its potential interaction with HLA-G has revealed no evidence of direct receptor–ligand binding (200). In view of this, and in the absence of functional data using non-preactivated uNK cells, hypotheses regarding the role of uNK-expressed KIR2DL4 remain unsubstantiated.

**Table 5:** **Uterine natural killer (uNK) cell receptors and respective trophoblast ligands in human and mouse**.

uNK cell receptor	Trophoblast ligand
**Human**	
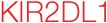 (A and B haplotypes)	Human leukocyte antigen (HLA)-C2 (116, 119)
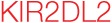 (B haplotype)	HLA-C1 (116, 119)
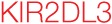 (A haplotype)	HLA-C1 (116, 119)
KIR2DL4 (A and B haplotypes)	HLA-G? (201)
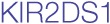 (B haplotype)	HLA-C2 (116, 119)
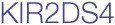 (A haplotype)	HLA-C1, HLA-C2 (202)
LILRB1	HLA-G (197, 203)
CD94:NKG2A	HLA-E (197)
CD94:NKG2C; CD94:NKG2E	HLA-E (197)
NKp44	Unidentified (4, 179)
NKp46	Unidentified (4, 204)
NKG2D	Not expressed (20)
**Mouse**	
Ly49A, Ly49G2	H-2D^d^ (BALB/c) (36)
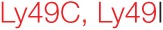	H-2K^b^ (C57BL/6) (37)
Ly49D	H-2D^d^ (BALB/c) (36)
Ly49H	Not expressed
CD94:NKG2A	Not expressed (205)
CD94:NKG2C; CD94:NKG2E	Not expressed (205)
NKG2D	Rae1 (44)

*Inhibitory receptors denoted in red, and activating receptors denoted in blue*.

*KIR2DL4 can be both inhibitory and activating*.

As in humans, murine trophoblast cells at the site of physiological exchange are MHC negative, whereas invasive trophoblast does express MHC class I antigens (36, 37, 206). Invasive TGCs from C57BL/6 mice have been shown to express H-2K^b^ ligands at far greater intensity than H-2D^b^, which is only detectable at very low levels (37). Trophoblast expression of transgenic H-2D^d^ epitopes in C57BL/6 mice has also been demonstrated by immunofluorescence staining (36). uNK cells interact with trophoblast class I MHC through Ly49 receptors (Table 5), but since trophoblast do not express Qa-1^b^, any functional modulation of uNK cells through NKG2A/C/E is most likely mediated by decidual stromal cells (205, 207).

## Trophoblast and uNK Cell Interactions in Disorders of Pregnancy

Immunogenetic associations between maternal KIR/fetal HLA variants and disorders of pregnancy show that combinations of a maternal *KIR AA* genotype and fetal *C2* epitopes are present at significantly higher frequencies in pregnancies complicated by pre-eclampsia (208). Extension of this work later showed that this increased risk of developing pre-eclampsia is highest when trophoblast *C2* epitopes are paternally inherited (209). An increased frequency of maternal *KIR AA* and paternally derived *C2* epitopes has also been observed in cases of RM (209, 210). There is an additional weaker correlation, between maternal *KIR AA* and FGR (209). Within the *KIR A* haplotype are two genes for inhibitory KIR, *KIR2DL1* and *KIR2DL3*, which encode receptors for C2 and C1 epitopes, respectively (Table 5). It may be reasonably considered that KIR2DL1/C2 interactions are particularly detrimental to uNK cell function, as binding between KIR2DL1 and C2 epitopes is stronger and more specific than that between KIR2DL3 and C1 epitopes (211). Furthermore, interaction between KIR2DL1 and its cognate HLA-C ligand significantly reduces production of chemokines and angiogenic factors by IL-2-activated uNK cells *in vitro* (4).

Conversely, the presence of the telomeric region of the *KIR B* haplotype (*Tel-B*) was shown to be protective against RM and pre-eclampsia, particularly when trophoblast expressed C2 epitopes (208–210). The *Tel-B* region contains *KIR2DS1*, which encodes an activating KIR that binds C2 epitopes (Table 5). Maternal *KIR2DS1* predisposes to high birth weights, above the 90th centile, when the fetus expresses paternally derived C2 epitopes (212). Interaction between uNK cell KIR2DS1 and C2-expressing target cells *in vitro* induces GM-CSF production, which enhances trophoblast migration in a Transwell assay (178). Taken together, these data strongly indicate that uNK cell activity is modulated through KIR/HLA interactions and further support the hypothesis that imbalance in uNK cell function, potentially leading to dysregulation of physiological processes essential to pregnancy, can lead to undesirable reproductive outcomes in humans.

Most studies investigating murine uNK cell function to date have assessed the contribution of uNK cell deficiency or uNK cell-derived factors to reproductive success. Only more recently have mouse models been used to examine the impact of more subtle variations in uNK cell activity, such as that mediated by parental MHC disparity. Allogeneic, paternally inherited MHC class I is sufficient in isolation to modulate uNK cell function, and to directly impact on spiral arterial remodeling and fetal growth (36, 37). BALB/c females mated with BALB.B males, which express the C57BL/6 H-2^b^ MHC allotype, exhibit enhanced decidual vascular remodeling and increased fetal growth (37). However, the underlying mechanisms for this remain unclear. Paternally derived trophoblast H-2D^d^ has been convincingly demonstrated to inhibit uNK cell function in C57BL/6 females. Reduced production of IFN-γ by uNK cells was mediated by inhibitory interactions between H-2D^d^ with Ly49A and Ly49G2, which resulted in incomplete spiral arterial remodeling and reduced fetal growth. Maternally expressed H-2D^d^ was also disadvantageous for vascular transformation and fetal growth, which suggests that murine uNK cell education does not confer protective benefits during pregnancy (36).

The relative impact of maternal NKR variability as a determinant of pregnancy outcome in mice has been less well investigated. *Ncr1*^−/−^ mice, which lack NKp46, exhibit impaired decidual vascular remodeling and disrupted angiogenesis, which suggests that NKp46-mediated activation of uNK is important for optimal reproductive outcome in mice (47). Expression of Ly49 receptors can also influence pregnancy, as Ly49 knockdown (Ly49^KD^) mice, in which Ly49s are expressed by only 50% of DX5^+^ and 6% of DBA^+^ uNK, exhibit subfertility, impaired angiogenesis, reduced vascular remodeling and, unexpectedly, enhanced fetal growth (213). However, given the incongruence of the fetal phenotype in this model, it is feasible that extraneous factors are contributing to the outcomes observed. As the transcriptionally silenced region in Ly49^KD^ mice spans approximately 10.2 megabase pairs (Mbp), encompassing the ~2 Mbp NKC region, the potential impact of genes irrelevant to NK and/or leukocyte function cannot be discounted (214, 215). In keeping with defective angiogenesis, VEGF expression within the decidua and MLAp was reduced in Ly49^KD^ females. The total concentration of IFN-γ within these tissues was unaffected but, as uNK function was not specifically assessed in this study, it is not possible to determine whether uNK dysregulation is responsible for the vascular phenotype observed (213). As such, the strongest data relating to the consequences of NKR-MHC interactions in murine pregnancy are from studies investigating parental MHC disparity.

## Concluding Remarks

Results from many studies using mouse models to date have substantiated data from human genetic association studies, which strongly suggest that reduced uNK cell activation is disadvantageous for reproductive outcome. However, it is apparent that the success of pregnancy depends upon a highly complex network of interactions between trophoblast, uNK cells, decidual stromal cells, and other decidual leukocytes. New technology, including improved mouse models, high-throughput genotyping, mass cytometry, and single-cell RNA sequencing should help to define the role of immune cells in pregnancy, including tissue NK cells and other ILCs in human and mouse uterus.

## Author Contributions

The manuscript was written by LMG and edited by FC.

## Conflict of Interest Statement

The authors declare that the research was conducted in the absence of any commercial or financial relationships that could be construed as a potential conflict of interest.

## Abbreviations

cNK, conventional natural killer cells; NKR, natural killer receptor; pbNK, peripheral blood natural killer cells; pNK, peripheral natural killer cells; trNK, tissue-resident natural killer cells; uNK, uterine natural killer cells.
